# Mechanisms Associated with *Trypanosoma cruzi* Host Target Cell Adhesion, Recognition and Internalization

**DOI:** 10.3390/life11060534

**Published:** 2021-06-09

**Authors:** Oscar Hernán Rodríguez-Bejarano, Catalina Avendaño, Manuel Alfonso Patarroyo

**Affiliations:** 1 Health Sciences Faculty, Universidad de Ciencias Aplicadas y Ambientales (U.D.C.A), Calle 222#55-37, Bogotá 111166, Colombia; oscar.rodriguez@udca.edu.co; 2 Animal Science Faculty, Universidad de Ciencias Aplicadas y Ambientales (U.D.C.A), Calle 222#55-37, Bogotá 111166, Colombia; cavendano@udca.edu.co; 3 Molecular Biology and Immunology Department, Fundación Instituto de Inmunología de Colombia (FIDIC), Carrera 50#26-20, Bogotá 111321, Colombia; 4 Health Sciences Division, Main Campus, Universidad Santo Tomás, Carrera 9#51-11, Bogotá 110231, Colombia; 5 Microbiology Department, Faculty of Medicine, Universidad Nacional de Colombia, Carrera 45#26-85, Bogotá 111321, Colombia

**Keywords:** *Trypanosoma cruzi*, adhesion, recognition, internalization

## Abstract

Chagas disease is caused by the kinetoplastid parasite *Trypanosoma cruzi*, which is mainly transmitted by hematophagous insect bites. The parasite’s lifecycle has an obligate intracellular phase (amastigotes), while metacyclic and bloodstream-trypomastigotes are its infective forms. Mammalian host cell recognition of the parasite involves the interaction of numerous parasite and host cell plasma membrane molecules and domains (known as lipid rafts), thereby ensuring internalization by activating endocytosis mechanisms triggered by various signaling cascades in both host cells and the parasite. This increases cytoplasmatic Ca^2+^ and cAMP levels; cytoskeleton remodeling and endosome and lysosome intracellular system association are triggered, leading to parasitophorous vacuole formation. Its membrane becomes modified by containing the parasite’s infectious form within it. Once it has become internalized, the parasite seeks parasitophorous vacuole lysis for continuing its intracellular lifecycle, fragmenting such a vacuole’s membrane. This review covers the cellular and molecular mechanisms involved in *T. cruzi* adhesion to, recognition of and internalization in host target cells.

## 1. Introduction

Chagas disease or American trypanosomiasis is a disorder caused by *Trypanosoma cruzi* (a kinetoplastid flagellate parasite); it is mainly transmitted in endemic areas by triatomine hematophagous insect species (*Triatoma*, *Panstrongylus*, *Rhodnius*) and can affect the cardiovascular, digestive and central nervous systems [1]. The disease is a significant public health problem in Latin America since it is related to one of the most frequently occurring causes of heart failure (up to 41% of the cases in endemic areas) [2] and the loss of around 752,000 working days due to premature deaths. USD 1–2 billion productivity losses have been recorded in some South American countries where the parasite and its influence have been described [1]. The World Health Organization (WHO) has classified it as being among the 20 “Neglected tropical diseases” [3], estimating that 7000 deaths worldwide every year are associated with Chagas disease, that 200,000 new Chagas disease-related deaths will occur during the next five years and that 7 million people are currently suffering from it, along with 180,000 new *T. cruzi* infections every year [4].

Chagas disease has an acute phase which is characterized by high parasitemia lasting around 2 months, sometimes accompanied by systemic symptoms, such as fever, malaise, headache, anorexia, diarrhea, myalgia, lymphadenopathy, hepatomegaly, splenomegaly, generalized or local edema, skin rash, hemorrhagic manifestations, jaundice, myocarditis, tachycardia, arrythmias, atrioventricular block and meningoencephalitis [5]. Around 30% of affected patients progress to the disease’s chronic phase where detectable organ damage occurs, mainly affecting the cardiovascular and digestive systems, resulting from a severe inflammatory immune response leading to irreversible cell damage. Chagas cardiomyopathy is the main cause of non-ischemic cardiomyopathy in Latin America and is characterized by diffuse myocarditis along with focal fibrosis in the heart’s apex and posteroinferior walls [6].

The *T. cruzi* parasite has a complex lifecycle involving vertebrate hosts; its behavior is that of an obligate intracellular pathogen which can manipulate host cell mechanisms and processes to enable invasion [7]. A triatomine vector acquires trypomastigotes from an infected mammalian host’s bloodstream by biting it; the ingested trypomastigotes then become transformed into epimastigotes in the host’s midgut. The epimastigotes reach the triatomine’s hindgut and become differentiated into metacyclic trypomastigotes which are excreted in the vector’s feces. Metacyclic trypomastigotes enter a mammalian host through its skin or the mucosa and begin to invade different types of nucleated cells.

Once the trypomastigotes have entered a target cell, they remain in the parasitophorous vacuole (PV) where they become lysed, thereby enabling the parasites to escape into the cytoplasm and become differentiated into intracellular amastigotes which become replicated in host cell cytoplasm for 3 to 5 days (12-h doubling time). The amastigotes then become transformed into trypomastigotes which destroy the host cell, becoming free/released to infect new cells and begin new replication cycles or hematogenous dissemination, remaining available for triatomine uptake [8,9].

The infectivity rate seems to vary among different triatomine species and it has been shown that wild vector species have the highest infectivity levels, although domestic species are also very important regarding parasite transmission. *T. cruzi* has been classified by molecular markers into seven discrete typing units (DTU): TcI, TcII, TcIII, TcIV, TcV, TcVI and Tcbat. It seems that this factor associated with parasite strain, triatomine species and environmental conditions influences the effect of *T. cruzi* on triatomines, modifying infectivity and Chagas disease pathogenesis [10,11].

Various infection mechanisms have been proposed (such as phagocytic invasion and lysosome-dependent or -independent invasion) and used in attempts to explain this particular mode of infection. It must be borne in mind that *T. cruzi* has a broad variety of strains when considering its adhesion to and invasion of host cells, it has different infecting forms during its lifecycle (amastigotes and trypomastigotes), there are trypomastigote varieties (metacyclic, bloodstream- and tissue culture-derived) and host cell types which can be invaded [12].

The WHO introduced a roadmap (2020) regarding neglected tropical diseases 2021–2030; its objectives related to Chagas disease were: verifying the interruption of domiciliary vector-borne transmission, verifying the interruption of transmission by transfusions, verifying the interruption of transmission by organ transplants, eliminating congenital Chagas disease and broadening anti-parasite treatment coverage by 75% regarding the population at risk [13].

Only two drugs have been authorized for treating Chagas disease to date: benznidazole and nifurtimox; these have been the mainstay for anti-parasitic treatment for many decades now, even though it must be borne in mind that their safety and efficacy profiles are not ideal. Nifurtimox has been prescribed for oral use in three or four daily dose schemes over a 60–90 day period; it has had variable rates of cure during the chronic indeterminate phase, ranging from 7–8% in adults to 86% in children under 14 years of age, and adverse effects such as anorexia, weight loss, neurological alterations, nausea, vomiting, fever and exanthema (occurring variably). By comparison, benznidazole has better tolerance, tissue penetration and apparent efficacy and has been used orally at two or three doses daily over a 60-day period. Benznidazole is used as first line of pharmacological treatment in the acute and early phase of Chagas disease, having 76–99% rates of cure; however, rates of cure range from 60–93% in the chronic phase in children under 14 years of age and 2–40% in adults, having adverse effects such as exanthema, digestive intolerance, anorexia, asthenia, headaches and sleep disorders (occurring variably) [1,3].

New therapeutic strategies must be developed for Chagas disease; studies have been carried out to such in animal models using many types of drugs already approved for other uses. Nifedipine, amlodipine, verapamil, flecainide, atenolol, procainamide, bisoprostol, pyrimethamine, defibrotide, gliclazide, enalapril, albendazole, artemisinin–piperaquine, meglumine, metformin, pentoxifylline, paromomycin, miltefosine, ivermectin, quinine, lidocaine, mebendazole, atovaquone, diltiazem, clioquinol, nicardipine, pentamidine, primaquine, artemether–lumefantrine, nadolol, sotalol and tinidazole activity has been proved against *T. cruzi*, but the results have been inconclusive [14,15].

Obtaining a vaccine that can control *T. cruzi* transmission and Chagas disease has become an urgent and relevant challenge. Significant research efforts associated with developing experimental vaccines have been made during the last few decades, leading to some promising results in animal models. Work has been performed on the search for vaccine candidates and selecting immunogens, adjuvants, DNA-based vaccines and designing therapeutic vaccines. However, the large amount of variables to be considered and the lack of uniform criteria among research groups have been significant limitations on obtaining a vaccine [16,17].

Some published reviews have focused on particular topics regarding *T. cruzi* infection; however, progress has been made during recent years regarding the understanding of the mechanisms related to parasite internalization in host cells. Different types of endocytosis occurring during the invasion of target cells by the parasite’s infectious forms have been described using experimental models and in greater molecular detail, although such mechanisms are still not entirely clear. This would include clathrin-mediated endocytosis, membrane microdomain-mediated endocytosis, macropinocytosis and phagocytosis. Some receptors associated with adhesion and parasite recognition on host cell membranes have been found for some trans-sialidase superfamily molecules from *T. cruzi* trypomastigotes, i.e., LAMP-2 and LDLR. This review aims at providing an update regarding the cellular and molecular mechanisms involved in *T. cruzi* adhesion to and invasion of host cells.

## 2. Molecules Involved in *Trypanosoma cruzi* Entry into Host Cells

*T. cruzi* studies have shown that the parasite has tropism for almost all phagocytic and non-phagocytic nucleated cells. Many cellular and molecular events involved in mammalian host cell infection are still not fully understood, though focused research has now clarified some of them. It is thus already known that key events in *T. cruzi* internalization in host cells are binding and adhesion to the plasma membrane (PM), triggering a specific signaling cascade activating mechanisms for parasite internalization and PV formation.

*T. cruzi*’s surface is covered by a dense glycocalyx whose composition is characteristic of each stage in the parasite’s lifecycle [18]. The nature of the carbohydrates on the parasite’s surface largely depends on unique changes in their composition during differentiation and lifecycle [19]. The most abundant and relevant parasite surface molecules are free glycoinositolphospholipids (GIPLs), along with the mucins which are glycoproteins bound to PM via a glycosylphosphatidylinositol (GPI) anchor [20,21].

Many molecules are involved in *T. cruzi* adhesion to host cell PM and invasion; they have been grouped into various families (Table 1).

### 2.1. The Mucins

*T. cruzi* is covered by a dense layer of mucin-type glycoproteins; these have carbohydrate residues which can interact with the host cell surface. These proteins are encoded by regions accounting for around 1% of the parasite’s genome and are characteristic and distributed in large amounts on the cell body, the flagellum and the flagellar pocket of different forms of parasite development [20]. These proteins consist of 50–200 amino acids, having a large amount of O-glycosylated serine and threonine residues; this makes the mucins highly hydrophilic [21]. As a large amount of these molecules are expressed in *T. cruzi* trypomastigotes and amastigotes, it is considered that they participate in cell adhesion and invasion; host immune responses suggest that they are also involved in the parasite’s immune system evasion mechanisms due to variation in the core of mucin-type polypeptides expressed during the parasite’s mammalian stages [22].

Taking their sequence identity into account, the genes encoding *T. cruzi* mucins have been classified into two families called TcMUC and TcSMUG [22,23]. The TcMUC genes encode three groups of mucins in terms of their central domains: TcMUC I and II are distributed on bloodstream amastigote and trypomastigote forms’ surface [24] and TcMUC III, which is called trypomastigote small surface antigen (TSAA) [25]. The TcSMUG gene encodes small (S) and large (L) groups of mucins, according to the size of their encoding mRNA. The S group is found in epimastigotes and metacyclic trypomastigotes and encodes N-glycosylated 35–50 kDa (Gp35/50) mucins, which are the main acceptors of the sialic acid transferred from the trans-sialidases on the parasite’s surface [26,27,28]. The L group encoding mucin-type glycoconjugates are not sialic acid acceptors and are only present on the parasite’s surface during epimastigote and metacyclic trypomastigote stages [29,30,31].

### 2.2. The Trans-Sialidase Superfamily

Like the mucins, the trans-sialidases (TS) are distributed all over the body, the parasite’s flagellum and flagellar pocket [57]. The TS superfamily is divided into four groups of proteins according to the identity of the sequence and such molecules’ functional properties [58]. Group I consists of proteins with trans-sialidase activity (sialic acid transfer from host cell glycoconjugates to trypomastigote mucins [32]) and/or neuraminidase activity (when acceptor molecules are not suitable for receiving sialic acid, it is released to the milieu) [33,34]. *T. cruzi* sialylation is a key event for its viability and propagation in host cells [59,60], and neuraminidase activity is a key element for parasite internalization [35,36]. *T. cruzi* neuraminidase (TCNA) and shed acute phase antigen (SAPA) are the TS Group I members in the bloodstream trypomastigote stage and epimastigote trans-sialidase (TS-epi) [31] in the epimastigote stage. Both SAPA and TCNA are GPI-anchored to the parasite’s PM, whereas TS-epi is not GPI-anchored; it has been predicted that the membrane anchoring of the latter is due to a transmembrane C-terminal domain [31,37].

Group II consists of members of the GP85 surface glycoprotein family, such as ASP-1, ASP-2, TSA-1, Tc85, SA85, GP82 and GP90, which have been associated with binding to and invasion of host cells and are GPI-anchored to the parasite membrane [38,39,51,61]. Amastigote surface proteins 1 and 2 (ASP-1 and ASP-2) and trypomastigote surface antigen 1 (TSA-1) represent specific targets for specific CD8+ lymphocytes during a cytotoxic response to *T. cruzi*, and it has been seen that they can intensely stimulate antibody (Ab) formation in infected mice and humans [7,31,38]. Glycoprotein surface antigen 85 (SA85) is expressed in bloodstream amastigote and trypomastigote forms [40]. *T. cruzi* 85 (Tc85, due to its 85 kDa molecular weight) occurs abundantly in bloodstream trypomastigotes and can bind to different host receptor molecules, i.e., some extracellular matrix ones (cytokeratin 18, fibronectin and laminin) and others located on monocyte, neutrophil or fibroblast cell surface [40,41,42,43]. GP82 and GP90 are glycoproteins expressed on the metacyclic trypomastigote surface [44,45]. GP82 can activate parasite internalization following its adhesion to host cells [40,46,47,48]. GP90 in metacyclic trypomastigote form lacks enzyme activity [48] but seems to be a negative regulator of parasite invasion, while having an antiphagocytic effect during bloodstream amastigote and trypomastigote stages; this is mediated by the elimination of the carbohydrate residues necessary for parasite internalization by possible glucosidase activity [8,49,50].

Group III is formed by surface proteins in bloodstream trypomastigotes, and includes CRP, FL160, CEA and TESA. These proteins can inhibit classical and alternative complement activation pathways, which could be a way of protecting the trypomastigote form against lysis by the host [8,51,52]. The trypomastigote excreted–secreted antigen (TESA) is distributed on the cell membrane, while the complement regulatory protein (CRP) provides the parasite with protection against host complement lytic activity. Flagellar 160 (FL160) and chronic exoantigen (CEA) proteins are membrane proteins associated with the trypomastigote flagellum [53,54,55]. Group IV consists of genes encoding trypomastigote surface antigens whose biological function remains unknown; a protein representative of this group would be the *T. cruzi* 13 protein (Tc13), which has been shown to be highly antigenic and is found in metacyclic trypomastigotes [56].

Although all the aforementioned *T. cruzi* ligands have been associated with phagocytic and non-phagocytic cell adhesion and invasion, it is still not clear what happens to each specific cell group following parasite adhesion [31].

## 3. Overall Steps for *Trypanosoma cruzi* Entry into Target

*T. cruzi* has a complex lifecycle, involving an obligate intracellular stage during which parasite replication occurs. It has been clearly established that gastric mucosa cells, cardiomyocytes, smooth myocytes and dendritic cells are the parasite’s target cells; however, little is known about other specific cell types which could be invaded in different tissues during acute and chronic *T. cruzi* infection. The parasite can infect most nucleated cell types in culture; however, associating this with different *T. cruzi* strains’ genetic and biological diversity and the parasite strain–host cell combinations which have been used has not resulted in a coherent universal cell model for studying parasite invasion.

**Figure 1:**
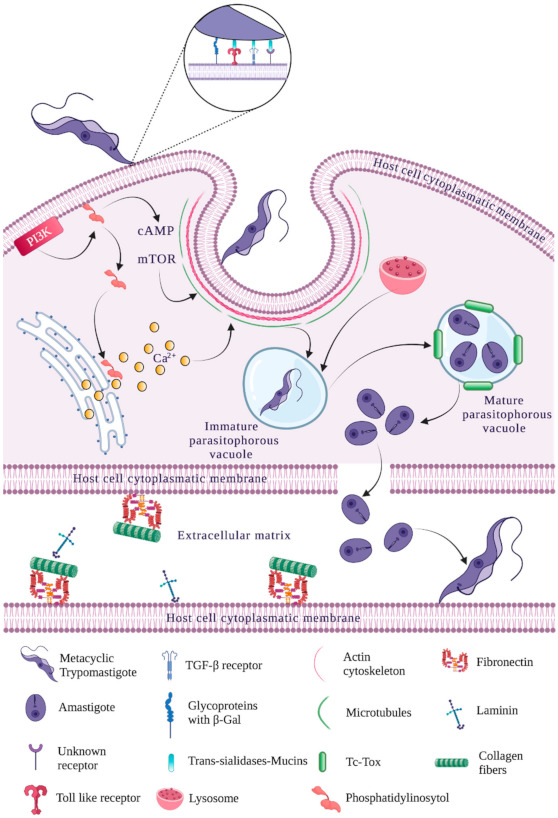
Mobilization, adhesion, recognition and internalization during each stage of *T. cruzi* invasion. Host cell recognition of the parasite involves the interaction of many molecules from both the parasite and host cell plasma membrane to achieve internalization via the activation of different endocytosis mechanisms. This is triggered by various signaling cascades, including PI3K, leading to increased Ca^2+^ and cAMP concentrations responsible for reordering cytoskeleton components and the association of endosomes and lysosomes to form the parasitophorous vacuole. The parasite fragments the parasitophorous vacuole’s membrane and continues its intracellular lifecycle so that it can escape towards the extracellular matrix and begin a new invasion cycle. Created with BioRender.com, accessed on 8 June 2021.

**Figure 2:**
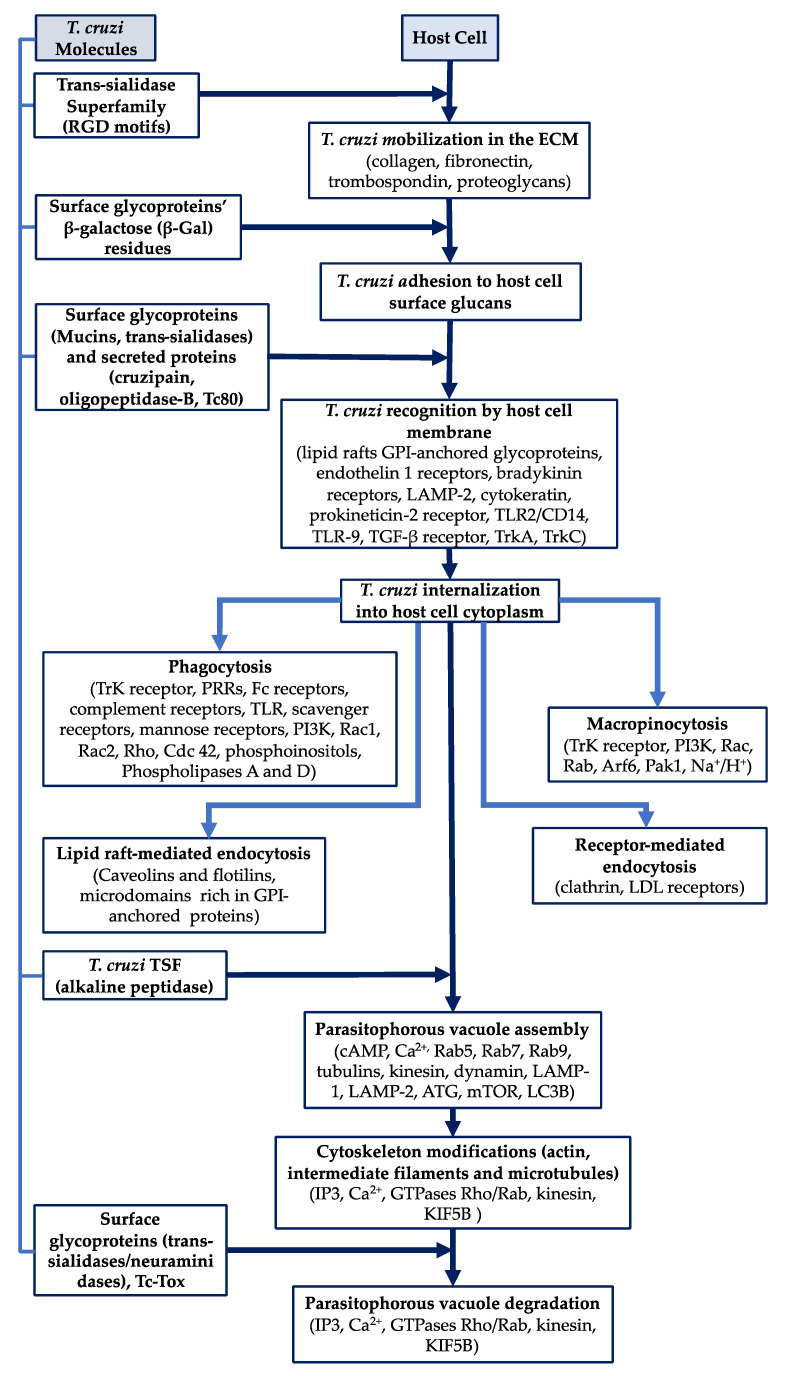
Processes and molecules associated with each mechanism involved in *Trypanosoma cruzi* invasion of host target cells.

Nevertheless, advances have been made regarding understanding host cell recognition mechanisms and *T. cruzi* trypomastigote signaling and invasion [62]. *T. cruzi* invasion of different host cells involves a series of complex interactions between the parasite, the extracellular matrix and target cells; these have been described as a series of steps called mobilization, adhesion, recognition and internalization (Figure 1). These steps and the cellular and molecular mechanisms involved in them have proved difficult to predict because the studies undertaken for such a purpose used different *T. cruzi* strains, the parasite development stage for studying the aforementioned events has varied, the trypomastigote form used has been modified (bloodstream and/or metacyclic) and the same host cell line type has not always been taken into account for the different assays [8,63]. A schematic representation of *T. cruzi* invasion of host target cells is shown in Figure 2, mentioning the main parasite and host cell molecules involved with each mechanism.

### 3.1. Mobilization in the Extracellular Matrix

*T. cruzi* trypomastigotes represent the parasite’s most specialized stage; they circulate in the blood, thereby presenting the likelihood of a great variety of tissues becoming infected. The trypomastigotes can bind to extracellular matrix components (EMCs) and use them for mobilizing towards host cells, this being an important characteristic regarding how *T. cruzi* infection becomes established [64]. Trypomastigote molecules from the trans-sialidase (TS) superfamily can bind to host EMCs such as laminin, collagen, fibronectin thrombospondin and heparan sulphate proteoglycans. Some members of the TS superfamily contain the RGD motif (arginine–glycine–aspartic acid) which has been characterized as an integrin binding domain [65]. Dispersed gene family-1 (DGF-1) superfamily members also contain RGD motifs and carbohydrate binding sequences, indicating their role in binding to EMCs [66].

### 3.2. Adhesion to Host Cell Membrane

The first step in parasite interaction with host cells requires the recognition of parasite surface molecules by their respective receptors on the host cell membrane. Some members of the Apicomplexa family are associated with a phenomenon mediated by molecules secreted by the parasite during this invasion phase, i.e., the cruzipain cysteine protease which is secreted in the flagellar pocket; various studies have shown that it mediates *T. cruzi* host cell infectivity through the cleavage of a high molecular weight kininogen, thereby producing short-lived kinins which bind to the bradykinin receptor, stimulating Ca^2+^ release mediated by inositol triphosphate (IP3), a very relevant event in parasite internalization by host cells [67,68,69].

Adhesion has been experimentally shown to occur at low temperatures (i.e., 4 °C) and can be inhibited by molecules such as cytochalasins, thereby avoiding actin polymerization; however, parasite adhesion to the host cell PM does not necessarily mean that it will be invaded [27]. It has been suggested that surface glycoproteins’ β-galactose (β-Gal) residues promote parasite binding and entry into host cells since it is thought that soluble lectin family galectins binding to glycoconjugates containing β-Gal (i.e., parasite surface glucans) can become associated with host cell membrane glucans to form a complex cell surface network for optimal receptor spacing and signaling [70,71,72].

### 3.3. Recognition by Host Cell Membrane

The key to *T. cruzi* trypomastigote survival is that they can come into contact with the host cell surface once they have become mobilized in the EMC, forming stable interactions prior to entering the cytoplasm via surface glycoproteins (mucins and trans-sialidases), binding to their cell surface receptors and avoiding protease action. Such molecules have many functions since they can be adhesins, destroy EMCs, modify ligands, help in avoiding the immune system or activate signaling cascades in both the parasite and host cells [65,73,74]. A very important event in parasite recognition consists of surface mucins becoming modified by the sialic acid eliminated from the host cell membrane by trans-sialidase action [59,75].

The presence of highly specialized regions or microdomains called lipid rafts is another important factor for parasite recognition as they coordinate and regulate cell signaling-associated events by temporally and spatially organizing membrane proteins for anchoring to the PM by the GPI motif (i.e., GPI-anchored proteins are highly concentrated in lipid rafts) [76]. Cell signaling triggered by coordinating events between surfaces and cholesterol eliminators via the lipid rafts affecting membrane fluidity and raft reorganization is also relevant for parasite recognition [77,78,79]. Regarding cardiovascular cells, other molecules associated with trypomastigote recognition have been described, such as endothelin 1 and bradykinin receptors [80]. The recognition of trypomastigote and amastigote parasite forms by the interaction of different surface molecules with the host cell membrane to enable invasion is discussed below.

#### 3.3.1. Trypomastigote

Trypomastigote-expressed trans-sialidases GPI-anchored to the parasite membrane have a catalytic N-terminal region and a C-terminal region containing SAPA tandem repeats. The trans-sialidases transfer sialic acid to trypomastigote PM mucins; such sialylation provides the parasite with complement resistance, this being important in invasion [60,81]. Host cell invasion by metacyclic trypomastigotes is mediated by a specific surface glycoprotein (gp82) which can activate a signaling pathway mediating tyrosine residue phosphorylation and becomes Ca^2+^-dependent in host cells following trypomastigote adhesion; this is necessary for continuing invasion and subsequent internalization since binding to its receptor induces lysosome dissemination and the exocytosis required for PV formation [39,45,46,82,83]. Recent experiments have provided data suggesting that metacyclic trypomastigote invasion is achieved by gp82 being recognized by its receptor, i.e., lysosomal associated membrane protein-2 (LAMP-2) [84,85].

Trypomastigote gp90 (appearing to have glucosidase activity) has a regulator effect mediated by the elimination of carbohydrate residues which are necessary for parasite internalization, thereby avoiding host cell invasion. This effect has been associated with a lack of stimulation of Ca^2+^ intracellular current necessary for internalization in both host cells and the parasite [27,82,86]. It has been shown that reduced gp90 expression on parasite surface conditions increased parasite invasion of host cells, suggesting that gp90 avoids gp82 being able to bind to its host cell membrane ligand, thereby negatively affecting parasite invasion [27,63,87,88].

It has also been proposed that gp90 plays a negative regulator role in trypomastigote internalization, this being mainly associated with this molecule’s ability to inhibit lysosomal dissemination within host cells [48]. gp90 has been shown to play a relevant role in *T. cruzi* infection by oral route and it has also been shown that the course of infection can vary depending on the gp90 isoform expressed in a determined parasite strain. Strains expressing gp90 susceptible to degradation by gastric pepsin at acid pH have been shown to have high gastric epithelial cell invasion rates, while strains expressing gp90 which are resistant to digestion by pepsin have been shown to have poor internalization [89,90]. gp90 thus plays a decisive role in modulating *T. cruzi* invasion of host cells.

Trans-sialidase Tc85 is also abundant in the trypomastigotes and forms a group of GPI-anchored glycoproteins having similar molecular weights but different isoelectric points (pI), which can become ligated to host cell membrane receptors such as cytokeratin 18 [65]. It has been shown that using monoclonal antibodies (mAbs) targeting Tc85 has inhibited host cell invasion rate by 50–96% [65,91,92]. The GGIALAG sequence is the region involved in Tc85 adhesion to cells; it is found in the prokineticin-2 receptor (PKR2) as a ligand for *T. cruzi* infection. The site for Tc85 binding to PKR2 is located in the molecule’s C-terminal extreme, upstream of the conserved FLY sequence which has been shown to be implicated in parasite invasion. PKR2 is a receptor which is formed by seven α-helix transmembrane segments and is mainly located in the central nervous system (CNS), the peripheral organs and mature blood cells. As PKR2 is widely distributed, it could be a suitable receptor for natural infection by trypomastigotes, contributing to parasite dissemination in mammals [93].

Glycoproteins called surface mucins have also been involved in the parasite recognition of host cells and their subsequent invasion since they interact with protein membrane surface receptors through the participation of their carbohydrate residues in parasite recognition and invasion of the host cell membrane. The mucins could trigger Ca^2+^ mobilization in host cells; this is directly related to the initial parasite invasion phase [22,24,82]. Metacyclic trypomastigote mucins have 35–50 kDa molecular weight (gp35/50) and Abs targeting their carbohydrate residues have been used to inhibit host cell invasion by the parasite [94]. gp35/50 mucins mainly found in CL strain trypomastigotes have been shown to be able to resist digestion by proteases and are responsible for protecting this parasite form during infection by oral route [90,95]. Mucins from host cell-derived trypomastigotes weigh 60–200 kDa after having completed the intracellular replication cycle; they all have the Ssp-3 epitope containing sialic acid which is crucial for cell adhesion and invasion and which could be involved in regulating the complement cascade [35]. These mucins’ O-oligosaccharides differ from those of insect stage mucins (epimastigotes) since they have terminal Gal(α1,3)Gal epitopes, which are the main target of the humoral immune response system [96]. The Gp83 glycoprotein is another ligand used by trypomastigotes for binding to and entering phagocytic and non-phagocytic cells [94,97].

Some parasite proteases, such as cruzipain, oligopeptidase-B and Tc80, have been associated with host cell invasion. Cruzipain is a highly mannosylated cysteine protease located in epimastigotes’ endosome–lysosome system and on the surface of epimastigotes and amastigote–trypomastigote transitional forms. It is secreted in the parasite’s flagellar pocket and has been described as cleaving high molecular weight kininogen in host cells, creating short-lived kinins which bind to the bradykinin receptor to stimulate IP3-mediated Ca^2+^ release, with such a pathway being associated with parasite internalization [67]. Oligopeptidase-B is a serine endopeptidase secreted by trypomastigotes and seems to indirectly induce a transitory increase in Ca^2+^ concentration during parasite invasion [98,99]. Tc80 is a prolyl oligopeptidase which hydrolyses fibronectin and collagen type I and IV and, more than being associated with parasite adhesion to and recognition of host cells, represents an important intermediary in parasite mobilization through EMCs [100].

Other studies have been carried out with many types of host cell; they have tried to link some molecules to parasite recognition; for example, invasion was blocked in cytochalasin B-treated macrophages and trypomastigote invasion became altered in Vero cells and muscle cells of chicken treated with concanavalin-A, phytohemagglutinin, wheat germ agglutinin, ricin, trypsin and periodate, also highlighting the participation of surface proteins and glycoproteins during invasion [8,101,102]. TLR-4 and TLR-9 are Toll-like receptors (TLRs) which recognize *T. cruzi*. It has been described that TLR2/CD14 acts as mucins’ GPI domain receptor and that GIPLs induce trypomastigote phagocytosis [103,104,105,106].

It has also been observed that TLR-9 is activated by CpG-rich methylated DNA and *T. cruzi* DNA in macrophages [105]. The TGF-β receptor facilitates the recognition of *T. cruzi* in epithelial cells; although the trypomastigote ligand molecule remains unknown, it has been suggested that a similar factor to TGF-β having a phosphatidylserine residue could be an activator for this receptor [107,108]. Endothelin-1 receptors are also used by trypomastigotes in recognizing cardiovascular cells and tissues submitted to some type of stress or lesion and are particularly important during trypomastigote invasion of cardiomyocytes and endothelial cells, probably conditioning Chagas’ cardio-vasculopathy development [80]. It has also been seen that the nerve growth factor tyrosine kinase-A (TrkA) receptor on neurons and dendritic cells participates in *T. cruzi* invasion through trypomastigote trans-sialidases [109], as does the tyrosine kinase C (TrkC) receptor on neurons and glial cells by the parasite-derived neurotrophic factor (PDNF) and the TS [110].

#### 3.3.2. Amastigote

Regarding the amastigotes, the surface mechanisms and molecules associated with adherence to and invasion of host cells are still not clear. A carbohydrate epitope and a 21kDa protein have been identified which could be implicated in amastigote recognition since they have inhibited amastigote invasion of targeted cells by being recognized by mAbs [111,112].

### 3.4. Internalization into Target Host Cells

Following parasite adhesion to and recognition of the host cell membrane, infection continues via parasite internalization in the cytoplasm. It has been suggested that internalization could occur when the parasite’s environmental temperature is higher than 18 °C [113]. Various mechanisms by which the parasite could become internalized have been postulated, such as phagocytosis and endocytosis, thereby enabling the parasite to enter host cells and become isolated in the cytoplasm within the PV, which protects it from lysosomal destruction while undergoing its intracellular replication cycle [114].

The molecules involved in endocytic mechanisms for parasite internalization have not all been described, but a premise has been advanced that this endocytosis pathway begins with parasite recognition of surface molecules by host cell membrane receptors (some mentioned previously). Endocytosis consists of a series of mechanisms, including the clathrin-independent endocytosis pathway, clathrin-mediated endocytosis, caveola mediated-endocytosis, phagocytosis, micropinocytosis and circular dorsal ruffle formation [115,116].

#### 3.4.1. Phagocytosis

Host cells must remodel the actin cytoskeleton (AC) so that they can emit pseudopod-type cytoplasmatic prolongations to ensure successful parasite internalization. It has been seen that phagocytic cells stimulate tyrosine kinases, thereby activating phosphatidylinositol—3 kinase, in turn participating in AC remodeling to enable these pseudopods to be emitted during trypomastigote phagocytosis [117]. Phagocytosis can be activated by various types of ligands and receptors; some associated with pathogens, known as pattern recognition receptors (PRRs), are found on phagocytic cells’ cellular membrane and are known as Fc receptors, complement receptors, Toll-like receptors, scavenger receptors, mannose receptors and EMC receptors [118,119].

The signaling so triggered depends on the chemical nature of the receptors used; once stimulated, they cause the phosphorylation of the tyrosine kinases responsible for activating AC remodeling. Some molecules such as Rac1, Rac2, Rho, Cdc 42 and the phosphoinositols (phosphatidylinositol-4,5-bisphosphate and phosphatidylinositol-3,4,5-triphosphate) participate following their activation in the actin remodeling during phagocytosis caused by pseudopod formation [117]. Phospholipases A and D have also been seen to be involved in phagocytosis [120]. It has been suggested that using cytochalasin-B with different cell lines (peritoneal macrophages, L929, HeLa and bovine fetal fibroblasts) can block AC remodeling and that trypomastigotes invade host cells using the phagocytosis mechanism [121].

Regarding cardiomyocytes, it has been shown that trypomastigotes induce phagocytosis by pseudopod formation [122,123]; other mechanisms (such as micropinocytosis, clathrin-mediated endocytosis and microdomains) have been included recently as they participate in the membrane constituted by lipid rafts and in the mechanisms associated with phagocytosis [115]. Amastigotes seem to have induced phagocytosis for non-phagocytic and phagocytic cells (macrophages) in the different strains which have been studied [124]. In vitro assays have demonstrated that amastigotes can also induce phagocytosis by remodeling the AC in experiments with cytochalasin D-treated host cells [125].

#### 3.4.2. Lipid Raft-Mediated Endocytosis

It has been proposed that caveolar lipid rafts could participate in endocytosis via their characteristic proteins, called caveolins 1, 2 and 3; these are found in many cell types and via flat lipid rafts by caveolin-associated proteins called flotillins 1 and 2 [126,127,128]. Cholesterol is another important component in lipid rafts and promotes caveolar structural organization [129]. It has been seen that these rafts are rich in GPI-anchored proteins and that such host cell membrane microdomains are involved in *T. cruzi* invasion of phagocytic and non-phagocytic cells and have been associated with cholesterol, even though it is not clear whether cholesterol is the molecule acting in parasite recognition or whether the amount of cholesterol in the microdomain is involved in the changes in membrane composition, causing modifications regarding the exposure of the pertinent receptors [77,79,130].

#### 3.4.3. Macropinocytosis

Macropinocytosis is characterized by the non-selective endocytosis of solute molecules, nutrients, antigens and some pathogens [131]. AC remodeling induces a stimulus triggering the activation of the tyrosine kinase receptors, thereby determining the emission of cell membrane ruffles. Receptor activation triggers some signaling pathways involving Rac1, Rab5, Arf6, PI3K and p21-activated Pak1 kinase [132].

Macropinocytosis likewise triggers the remodeling of the AC underlying the host cell PM along with the subsequent formation of vesicles called macropinosomes having different forms [133]. Both *T. cruzi* amastigotes and epimastigotes can use PI3K, Rac and Cdc 42 signaling pathways for invading different types of phagocytic and non-phagocytic host cells. It has been observed that Na^+^/H^+^ interchangers play a relevant role in macropinocytosis, since blocking them in host cells inhibits parasite internalization while stimulating them increases it [134].

#### 3.4.4. Receptor-Mediated Endocytosis (Clathrin-Mediated Endocytosis)

Clathrin-covered vesicles are formed by the self-assembly of clathrin heavy chain trimers associated with light chains. Depending on the type of vesicle content and the adaptor proteins and accessories necessary for vesicle assembly, a signaling pathway is produced using vesicle content for activating a determined cell mechanism [135]. Tyrosine kinase receptors, G protein coupled receptors, transferrin receptors, low-density lipoprotein (LDL) receptors and anthrax toxin receptors all participate in receptor-mediated endocytosis [136].

It has been seen that receptor-mediated endocytosis and AC remodeling are necessary for the internalization of microorganisms such as fungi, bacteria and viruses [115]. Experimental studies have led to observing that LDL lipoprotein receptors (LDLR) are very relevant for invasion and PV (containing *T. cruzi*) fusion with host cell lysosomes, suggesting that clathrin-mediated endocytosis is a parasite internalization mechanism via LDLR [137].

It has been shown experimentally that using clathrin-covered vesicle formation inhibitors (hypertonic medium containing saccharose, chlorpromazine hydrochloride and pitstop 2) and small interference RNA (siRNA) has significantly reduced *T. cruzi* trypomastigote and amastigote internalization in macrophages and epithelial cells. These recent assays have shown that clathrin is found around the parasites from the initial moment of parasite internalization and persists until PV formation, further supporting the idea that clathrin-mediated endocytosis participates in *T. cruzi* internalization in host cells [138].

### 3.5. Parasitophorous Vacuole Assembly

PV assembly is produced once the parasite has been recognized by host cells followed by consequent internalization by one of the previously mentioned pathways; the parasite modulates this by regulating PV membrane composition. Rapid lysosome recruitment is required for forming the PV containing *T. cruzi*; the lysosomes bind to the parasite following its internalization and become mobilized towards this meeting point using the microtubule cytoskeleton and mobilization molecules such as kinesin [10,139].

The host cell PM and lysosome membrane are required for PV formation and the regulation of membrane fusion, cytoplasmatic vesicle trafficking, microtubule reorganization, motor molecule activation, and calcium (Ca^2+^) and cyclic adenosine monophosphate (cAMP) signaling [140,141]. It has been suggested that early and late endosomes could also be sources of membrane for the PV [142,143]. Early endosomes contain molecular markers in their membranes such as Rab5 and early endosomal antigen-1 (EEA1) as well as an aqueous medium with a pH between 6.0 and 6.5 [144]. These organelles mature and become transformed into late endosomes which modify their main molecular marker Rab5 to Rab7, as well as being characterized by the appearance of other markers such as Rab9, Cd63 and the mannose 6-phosphate receptor. They can become mobilized in the cytoplasm by perinuclear microtubules. LAMP-1 and LAMP-2 proteins protecting acid hydrolases in late endosomes are also acquired during maturation through lysosome fusion in aqueous medium at 4.5 to 5.0 pH [145].

Early and late endosome participation in the interaction with the PV containing *T. cruzi* includes the dynamin-mediated Rab5–Rab7 signaling pathway [143,146]. It has been seen experimentally that around 20% of the vacuoles containing *T. cruzi* in host cells have endosomal markers (EEA1 and Rab5) and close to 20% have lysosomal markers (LAMP-1) [142]. Lysosomal fusion leads to PV acidification, which is fundamental for the *T. cruzi* intracellular replication cycle to occur [147]. Another essential event in PV assembly concerns parasite internalization in host cells inducing a signaling cascade, causing a bidirectional increase in intracellular calcium concentration which is responsible for cytoskeleton reorganization. This in turn is enabled via selected donors’ membrane movement and fusion and the appearance of the PV close to the parasite’s cell membrane invagination site [139,148,149,150].

Some assays have shown lysosomes’ ability to respond rapidly to an increase in intracellular calcium for regulating fusion with the PM, being important for the endocytosis pathway and the PM repair pathway regulated by intracellular calcium levels [151,152]. It has also been observed that a 120 kDa alkaline peptidase called *T. cruzi* TSF could induce repetitive calcium pulses from smooth endoplasmic reticulum in host cells during PV formation, and it seems that this is associated with sarcoplasmic reticulum calcium ATPase (SERCA) regulation of cardiomyocytes [153].

PV formation has also been associated with the autophagy pathway involving the recycling of degraded lysosomal organelle membranes; phagosomes can also be formed by this pathway [154,155]. Autophagy plays an important role in eliminating intracellular pathogens such as *T. cruzi* since the intracellular membranes envelop the organelles and cytoplasmatic residues, and these can be used for enveloping intracellular microorganisms in a phagosome (autophagosome) [156]. This involves proteins such as autophagy-related (ATG) proteins, the mechanistic target of rapamycin (mTOR) protein and the light chain of the microtubule-associated protein 3 (LC3B) [157]. Some assays have shown that autophagy is associated with *T. cruzi* internalization in host cells and that the PV contains LC3B as an autophagy pathway molecular marker [83,154]. Other studies have shown that the PV membrane also contains Fc receptors, β1 integrin, lysosomal membrane glycoproteins, complement receptors (CR3) and glycoconjugates [8,158,159].

### 3.6. Modifications to Host Cell Cytoskeleton

*T. cruzi* host cell cytoskeleton components are extremely relevant for parasite invasion to be successful. The actin filaments must become reorganized during a calcium-mediated process to facilitate parasite internalization [140,142,160]. The behavior involved in reorganizing the actin filaments is not the same for all host cells and seems to depend on the parasite’s infective stage, i.e., trypomastigotes do not modify the AC in the same way as the amastigotes do [125]. Some other molecular elements associated with the AC in some way or other have been seen to undergo modification or reorganization during parasite internalization in host cells, such as the intermediate filaments, the myosin-related apparatus, the integrins and EMCs [124]. GTPases Rho/Rab are key elements in cytoskeleton modification via a signaling cascade during host cell surface recognition and subsequent parasite internalization; however, these GTPases’ signaling cascade is not the same, depending on the infective form (trypomastigote or amastigote), since, for example, signaling mainly depends on Rac1 during amastigote invasion [143,161].

It has been seen that pharmacologically blocking actin polymerization increases trypomastigote entry into non-professional phagocytic cells; the same does not occur with the phagocytic capture of latex beads or bacteria in the same cells. Exposing these cells to trypomastigotes triggers an increase in intracellular IP3-dependent Ca^2+^ levels, along with the subsequent rapid and transitory reorganization of the AC [149,162,163]. It has been proposed (not altogether clearly) that the AC acts as a barrier to avoid lysosome coupling to and fusion with the PM and that the cytoskeleton’s transitory depolymerization with increased transitory Ca^2+^ concentrations leads to a greater possibility of lysosome fusion with the PM [142,149,162,163].

The microtubules also play a key role in *T. cruzi* invasion of target host cells [139,164]. Experiments have shown that pharmacologically blocking microtubule dynamics stability by their interaction with host cell tubulins reduces parasite invasion of non-phagocytic cells, such as fibroblasts and myocytes [139]. Microtubule modifications participating in the mobilization of endosome compartments during parasite internalization mainly depend on changes in cytoplasmatic calcium concentrations caused by the parasite entering host cells [164].

It is thought that an attraction of γ-tubulin units occurs during PV formation, enabling the PV to adhere to nearby microtubules in a relatively passive manner rather than an active one. It has also been proposed that alterations in host cell microtubule dynamics during parasite invasion apparently involve de novo microtubule polymerization, explaining the rapidity of lysosome transport to the trypomastigote entry site [164]. Changes in host cell cytoplasm pH cause an increase in lysosome movement towards the periphery caused by the microtubules with their motor proteins, such as kinesin, resulting in increased parasite invasion [160,165]. It has also been observed that blocking the kinesin heavy chain (KIF5B) inhibits *T. cruzi* internalization; KIF5B forms part of the microtubules’ positive extreme motor protein complex and is associated with lysosome transport and autophagosome formation [160,166].

### 3.7. Parasitophorous Vacuole Degradation

No matter which mechanism is used by *T. cruzi* trypomastigotes for their internalization in host cells, the parasite will become introduced into the PV and will seek a way to escape to the cytoplasm, which involves the degradation, disintegration or lysis of the PV membrane. This step is a key element in the *T. cruzi* intracellular lifecycle; however, it has not been fully characterized in studies to date.

The hypothesis was advanced (before any type of molecular identification) that *T. cruzi* could produce a pore-forming molecule capable of inserting itself into the PV membrane and begin its destruction. Initial studies showed that both *T. cruzi* trypomastigotes and amastigotes secreted and released a hemolysin with optimal activity at 5.5 acid pH, thereby being proposed as an excellent candidate for the PV’s supposed lysis activity [167]. Such a hemolysin was called Tc-Tox and liquid column chromatography showed that it became fractionated together with a 60 to 75 kDa protein which has a crossed reaction with Abs targeting complement component C9. Abs targeting Tc-Tox have been shown to be located in the Golgi apparatus membrane, the parasite’s flagellar pocket and infected cells’ phagosome membrane [168].

It has been observed that trypomastigotes release trans-sialidase/neuraminidase proteins within the PV and can eliminate sialic acid residues from the membranes; they can eliminate them from the PV membrane with subsequent sensitization regarding Tc-Tox peptide action, which is previously activated by the PV’s acid pH and apparently begins PV membrane destruction by forming pores [168,169,170]. Such a hypothesis has been supported by data from some assays where host cell cytoplasmatic pH has been pharmacologically increased, thereby delaying PV membrane degradation and indicating that an optimum pH is required for this. Deficient host cells have been used in sialylation in other assays in which it has been observed that the absence of sialic acid has made the PV membrane more sensitive to degradation. This has led to the hypothesis that sialic acid residues may play a regulator and protector role in lysosome membrane lysis [171].

It has also been suggested that Tc-Tox is active during the intracellular amastigote parasite stage, is invasive of phagocytic and non-phagocytic cells and could use the trans-sialidases for degrading the PV membrane more quickly than the trypomastigote form can do [114,124,172]. It has also been seen that pharmacologically increasing host cell cytoplasmatic pH has not modified the time amastigotes spend in the PV regarding base conditions [171,172]. Trypomastigotes initiate differentiation in amastigotes while still inside the PV and even during its membrane fragmentation [172].

## 4. Conclusions and Perspectives

Chagas disease, or American trypanosomiasis, continues being an entity affecting millions of human beings, especially those inhabiting Latin America. In spite of many efforts having been made for decades now aimed at obtaining vaccine candidates and therapeutic agents for fighting *Trypanosoma cruzi*, no vaccine has yet been approved for mass use on the population, nor has an innovative pharmacologic candidate been found against the etiological agent which meets the standards of quality, efficacy and safety for its clinical use.

Mammalian host cell recognition of the parasite’s infective form involves a molecular interaction between plasma membranes via the microdomains, thereby triggering some signaling cascades to enable parasite internalization by endocytosis. This is important due to the increase in Ca^2+^ and cAMP cytoplasm concentration, along with subsequent cytoskeleton reordering and PV formation whose membrane components become modified by the presence of the parasite’s infective form. The parasite takes advantage of PV lysis and continues its intracellular lifecycle.

This review has provided a detailed description of the different cellular and molecular mechanisms involved in parasite adhesion, recognition and internalization in human host cells; however, such mechanisms are still not entirely clear, thereby further hampering the design and development of therapeutic agents and/or vaccine candidates. Although some molecular targets have been identified on the parasite’s surface (such as the aforementioned trans-sialidase and mucin families and some host cell surface receptors that have been characterized), a deep understanding of their interaction with and participation in parasite adhesion and invasion is lacking; such knowledge would broaden the possibilities for discovering new therapeutic agents for combating this disease which is endemic to the Americas.

The reproducibility of assays worldwide has not been constant since the different groups working on developing vaccine molecules and/or therapeutic agents try to standardize their own protocols regarding parasite culture and infection in host cells when seeking to characterize such key molecules. Furthermore, such efforts are accompanied by the limitation of not all research groups using the same parasite strains; likewise, there is variability regarding the parasite stage used in the models (amastigotes, metacyclic trypomastigotes or bloodstream trypomastigotes). However, if one takes the large amount of strains described to date into account, along with the extensive repertoire of this parasite’s host cells in humans, then the difficulty of obtaining therapeutic agents against Chagas disease can be appreciated.

A considered recommendation would thus be to increase knowledge regarding adhesion and related invasion mechanisms and their variation regarding the different strains and host cells used in assays, thereby enabling greater understanding of the molecules involved in such processes for continuing in the race to develop a vaccine or specific pharmacological agent contributing towards worldwide control of this parasite. The WHO’s 2020 roadmap outlined objectives related to Chagas disease, i.e., interrupting vectoral transmission due to transfusions and organ transplants, eliminating congenital disease and broadening antiparasitic treatment coverage for the population at risk by 75%, posing significant challenges since ideal treatment is still not yet available.

Benznidazole and nifurtimox have mainly been used as drugs during the disease’s acute phase since 1960 as they have had variable effectiveness against *T. cruzi* extracellular forms in this phase; however, their effect has not been the best against the parasite’s intracellular forms found during indeterminate and chronic phases. Adverse effects and long-lasting treatment periods having variable efficacy have made their administration controversial. Scientific evidence regarding these drugs’ efficacy has led to them being included in treatment during the chronic phase for adults who do not have advanced cardiomyopathy and also in treating children who have acquired the infection congenitally.

Alternative drugs to benznidazole and nifurtimox from various chemical families have been studied; however, only some have completed preclinical phase studies with relative success because many of them have been shown to induce resistance in the parasite. Throughout this review, it has been suggested that selectivity regarding drug action must be considered when designing ideal trypanocidal agents. This must act on intracellular and extracellular forms, based on blocking parasitic molecules from the trans-sialidase superfamily and the mucin family as these are relevant during different invasion phases, such as mobilization in the extracellular matrix, parasitic adhesion and internalization in a host.

It has been shown how trans-sialidases induce signaling cascade activation, triggering the endocytosis of extracellular forms so that the intracellular replication cycle can be avoided by pharmacologically inhibiting such forms. Such a drug must act quickly to prevent the disease’s evolution to the chronic phase, its pharmacodynamics must enable reaching effective concentrations in plasma and body tissues, it must not induce parasite resistance, its toxicity and adverse effects must be minimal and it must be low cost and easily available to the population. Greater in-depth knowledge regarding receptors for parasitic trans-sialidases must be acquired since characterizing them better will enable the design of drugs selectively performing a competitive blockade. The recent discovery of LAMP-2 as a receptor for GP82 (a key trans-sialidase in parasitic adhesion to host cells) opens up the possibility of designing a pharmacological agent aimed at preventing this phase of parasite invasion.

High costs are involved in new drug production; forces must thus become joined and multidisciplinary and multinational initiatives promoted to make advances regarding the future challenge of obtaining an ideal drug for Chagas disease treatment.

Another important challenge concerns obtaining a vaccine that can control *T. cruzi* transmission. Significant efforts have been made during recent decades regarding research into vaccine development, including molecular targets mentioned in this review such as trans-sialidases or parasite-secreted molecules (i.e., cruzipain) which have shown promising results in animal models. Work has been performed on various vaccine candidates and selecting immunogens, adjuvants, DNA-based vaccines and designing therapeutic vaccines. The large amount of variables that must be considered and the lack of uniform criteria among research groups are limitations to obtaining an effective vaccine because (as suggested regarding drug development) multidisciplinary and multinational efforts and initiatives are required to make advances in finding a vaccine against Chagas disease.

## Figures and Tables

**Table 1:** Molecules associated with *Trypanosoma cruzi* entry into host cells.

Molecular Group	Subgroup	Parasite Stage	Members	References
Mucins	TcMUC	A and BT	TcMUC I	[22,23]
		A and BT	TcMUC II	[24]
		BT	TcMUC III (TSAA)	[25]
	TcSMUG	E and MT	TcSMUG S	[26,27,28]
		E	TcSMUG L	[29,30,31]
Trans-sialidase superfamily	TS I	BT	TCNA	[32,33,34]
		BT	SAPA	[35,36]
		E	TS-epi	[31,37]
	TS II	A	ASP-1 and ASP-2	[7,31,38,39]
		BT	TSA-1, Tc85 and SA85	[31,40,41,42,43]
		MT	GP82	[40,44,45,46,47,48]
		A, BT and MT	Gp90	[44,48,49,50]
	TS III	BT	CRP, FL160, CEA and TESA	[8,51,52,53,54,55]
	TS IV	MT	Tc13	[56]

E: epimastigote, A: amastigote, MT: metacyclic trypomastigote, BT: bloodstream trypomastigote.
